# Human Brain Activity Related to the Tactile Perception of Stickiness

**DOI:** 10.3389/fnhum.2017.00008

**Published:** 2017-01-20

**Authors:** Jiwon Yeon, Junsuk Kim, Jaekyun Ryu, Jang-Yeon Park, Soon-Cheol Chung, Sung-Phil Kim

**Affiliations:** ^1^Brain-Computer Interface Lab, Department of Human Factors Engineering, Ulsan National Institute of Science and TechnologyUlsan, South Korea; ^2^Department of Brain and Cognitive Engineering, Korea UniversitySeoul, South Korea; ^3^Center for Neuroscience Imaging Research, Institute for Basic ScienceSuwon, South Korea; ^4^Department of Biomedical Engineering, Magnetic Resonance Advanced Imaging Research Lab, Sungkyunkwan UniversitySuwon, South Korea; ^5^School of Biomedical Engineering, Konkuk UniversityChungju, South Korea

**Keywords:** fMRI, neural correlates, human perception, tactile, stickiness

## Abstract

While the perception of stickiness serves as one of the fundamental dimensions for tactile sensation, little has been elucidated about the stickiness sensation and its neural correlates. The present study investigated how the human brain responds to perceived tactile sticky stimuli using functional magnetic resonance imaging (fMRI). To evoke tactile perception of stickiness with multiple intensities, we generated silicone stimuli with varying catalyst ratios. Also, an acrylic sham stimulus was prepared to present a condition with no sticky sensation. From the two psychophysics experiments–the methods of constant stimuli and the magnitude estimation—we could classify the silicone stimuli into two groups according to whether a sticky perception was evoked: the *Supra-threshold* group that evoked sticky perception and the *Infra-threshold* group that did not. In the *Supra-threshold* vs. *Sham* contrast analysis of the fMRI data using the general linear model (GLM), the contralateral primary somatosensory area (S1) and ipsilateral dorsolateral prefrontal cortex (DLPFC) showed significant activations in subjects, whereas no significant result was found in the *Infra-threshold* vs. *Sham* contrast. This result indicates that the perception of stickiness not only activates the somatosensory cortex, but also possibly induces higher cognitive processes. Also, the *Supra- vs. Infra-threshold* contrast analysis revealed significant activations in several subcortical regions, including the pallidum, putamen, caudate and thalamus, as well as in another region spanning the insula and temporal cortices. These brain regions, previously known to be related to tactile discrimination, may subserve the discrimination of different intensities of tactile stickiness. The present study unveils the human neural correlates of the tactile perception of stickiness and may contribute to broadening the understanding of neural mechanisms associated with tactile perception.

## Introduction

Although people commonly place less importance on tactile than visual or auditory senses (Goldstein, 2013), the sense of touch is crucial for humans in interacting with the environment. It has been shown that tactile senses play an important role in recognizing physical properties of an object and allowing precise object manipulation through feedback information (Augurelle, 2002; Monzée et al., 2003; Nowak et al., 2003). Moreover, atypical tactile sensitivity is closely related with deficits in social interaction ability (Marco et al., 2011). A recent finding that people can even discriminate nanoscale textures suggests how exquisite the human tactile perception can be (Skedung et al., 2013).

To extend our understanding of the elaborate aspects of touch, many efforts have been devoted to finding fundamental dimensions of tactile perception. Previous works on the perceptual dimensions of tactile textures suggest 2–5 distinctive dimensions (Hollins et al., 2000; Ballesteros et al., 2005; Yoshioka et al., 2007; Tiest, 2010; Okamoto et al., 2013; Bensmaia, 2015). For example, Ballesteros et al. (2005) investigated the perception of 20 ecological surface texture stimuli by employing free classification and spatial arrangement tasks and grouped the stimuli into two perceptual dimensions (“roughness/smoothness” and “slippery/adherent”). Hollins et al. (2000) investigated the tactile texture perception of 17 different materials using the multidimensional scaling (MDS) method and constructed three fundamental dimensions representing “Rough/Smooth”, “Soft/Hard” and “Sticky/Slippery”. In addition to those three dimensions, other psychophysical studies have suggested “warm/cold” as another fundamental perceptual dimension (Tiest, 2010; Bensmaia, 2015). Moreover, Okamoto et al. (2013) proposed five fundamental dimensions for tactile perception, which subdivided the “roughness” in a more detailed way: macro roughness (uneven, relief), fine roughness (rough/smooth), warmness (warm/cold), hardness (hard/soft) and friction (moist/dry and sticky/slippery). Taking a consensus of those previous findings, tactile sensation can generally be classified in four fundamental dimensions: roughness, hardness, stickiness and warmness.

To date, a number of studies have investigated neural information processing underlying human tactile perception in each of those dimensions. Of the four dimensions, the roughness dimension has been the most extensively studied (Bensmaia, 2015). It is known that various mechanoreceptive afferents such as slowly adapting afferents (SAs), rapidly adapting afferents (RAs) and Pacinian afferents (PCs) contribute to roughness perception (Sathian et al., 1989; Blake et al., 1997). Neural substrates for roughness perception in the human brain were also explored. In a functional magnetic resonance imaging (fMRI) study (Kim et al., 2015), the primary somatosensory area (S1), supplementary motor area (SMA) and bilateral temporal poles were found to be responsible for perceptual sensitivity to tactile roughness. A considerable portion of neural mechanisms for the sense of warmness and hardness have also been unveiled. In thermal perception, the transient receptor potential (TRP) ion channels are known for transferring thermal information from object surfaces to the skin (Voets et al., 2004; Schepers and Ringkamp, 2010). Furthermore, Olausson et al. (2005) has demonstrated a relationship between the neural activity of the anterior insula cortex and subjective intensity ratings of warm feelings, suggesting that the insula plays an important role in perceiving thermal sensation. In the case of hardness perception, SA1 fibers are presumed to be responsible for perceiving hard and soft feelings from the surface of an object (Srinivasan and LaMotte, 1995; Bensmaia, 2015) and the postcentral gyrus, especially the Brodmann (BA) areas 3b and 1, is thought to contribute to perceiving hardness (Randolph and Semmes, 1974; Srinivasan and LaMotte, 1995; Servos et al., 2001).

On the contrary, relatively little has been known about the stickiness dimension and its neural mechanism. The tactile sense of stickiness is evoked when the skin is stretched during the detachment of a part of the body from an adhesive substance (Yamaoka et al., 2008). Yet, the mechanoreceptive afferents in charge of stickiness perception still remain controversial. On one hand, it is known that slowly adapting type 2 (SA2) afferents ending in Ruffini corpuscles are 2–4 times more sensitive to skin stretching than other afferents, and thus can process the information relating to skin stretching more effectively (Olausson et al., 2000; Johnson, 2001; Hale and Stanney, 2004). On the other hand, a number of studies reported that RA and SA1 afferents were more activated than other afferents in response to skin stretching (Johansson and Westling, 1987; Westling and Johansson, 1987; Srinivasan et al., 1990; Birznieks et al., 2001; Konyo et al., 2008). This inconsistency might in part stem from the use of a frictional force for creating the effect of skin stretching. To date, most studies on the perceptual mechanisms of stickiness have utilized the tangential movement of fingers (Srinivasan et al., 1990; Birznieks et al., 2001; Provancher and Sylvester, 2009) or grip (Johansson and Westling, 1987; Westling and Johansson, 1987) on the surface of an adhesive substance to evoke a sticky sensation. However, creating friction between the finger and a substance is naturally accompanied by other irrelevant factors such as direction and vibration (other than skin stretching) and thus hinders our ability to examine the sole effect of stickiness on tactile perception. Moreover, stickiness evoked by the frictional force is quite distant from its basic concept; the definition of the word “sticky” is interchangeable with “adhesive” or “viscous” (Merriam-Webster, 2011) but clearly distinguished from “nonslip.” The stickiness perception due to a frictional force is more of a “nonslip”, rather than a “stickiness”, and therefore, in a strict sense, experiments employing gripping or tangential movement may not properly measure neural responses generated by the perception of stickiness.

The present study was aimed at finding neural correlates of the tactile perception of stickiness in humans using fMRI. In particular, we focused on finding neural activity related to the “sticky” feeling, not a “nonslip” feeling. To achieve this, we prepared a set of silicon stimuli with varying levels of stickiness, which does not require the frictional force via the tangential finger movement in order to evoke sticky feelings. The aim of this study was pursued through two steps: psychophysical and fMRI experiments. In the first step, two psychophysical experiments were conducted to investigate the perception of stickiness evoked by the silicone stimuli: (1) the method of constant stimuli to measure an absolute threshold of the stimulus in a series of silicone stimuli; and (2) the magnitude estimation to measure the perceived intensity of stickiness (Goldstein, 2013). In the second step, an fMRI experiment with an event-related design was performed to explore brain regions associated with the stickiness perception. For data analysis, we used a general linear model (GLM) along with contrast analysis to identify the brain regions that showed activation when subjects perceived stickiness. Upon finding such regions, we investigated how the neural responses in these regions varied with the perceived intensity of the sticky sensation.

## Materials and Methods

### Participants and Ethics Approval

Twelve healthy all right-handed volunteers participated in the study (5 females, average 24.6 ± 2.47 years old, age range: 20–29 years old, excluding outliers). Participants had no history of neurological disorders or deficits. The study was approved by the ethical committee of Ulsan National Institute of Science and Technology (UNISTIRB-15-16-A) and all participants gave their written informed consent. As participants arrived at the experimental room, they were asked to read the MRI screening form carefully and asked to check items that he or she is relevant. After confirming that the participant is appropriate for the MRI experiment, an experimenter explains the object of the study and experimental procedure. Participants were also given sufficient time for reading the consent form and asking any questions. They also informed that they can cease the experiment at any time, even though they signed on the consent form. No vulnerable populations were involved in this study.

### Stimuli

Since the selection of sticky stimuli was crucial to this study, we made silicone-based sticky stimuli, polydimethylsiloxane (PDMS), designed to induce different intensities of stickiness in human perception. The material is used for developing a dry-adhesive system inspired by the attachment pad of gecko lizards (Yi et al., 2014a,b). By mixing the fast catalysts (CA-5275, GT Products Inc., TX, USA) into the liquid silicone (GT5727, GT Products Inc., TX, USA) with different ratios (5%, 6%, 7%, 8%, 9%, 10%, 15%, 20% and 30%;), PDMS stimuli with different intensities of stickiness were produced. The standard mixing ratio of the silicone and the catalyst was 10:1 (i.e., 10%). The lower the catalyst ratio was, the less the silicone was hardened, which made the substance stickier. The silicone products were cured in petri dishes (10035, SPL Life Sciences, Gyeonggi-do, South Korea) for a day after they were deflated in a vacuum chamber (OV-11, Jeio Tech.co, Daejeon, South Korea). They were then formed into cylinder shapes with 35-mm diameter and 5-mm height. Also, to make a sham stimulus with no stickiness, we cut a clear acrylic sheet into the same shape and size of the silicone stimulus. Each stimulus was attached to the corner of an 80 × 50 mm^2^ sized acrylic board. The board was used to allow the experimenter to lift the stimuli easily and to avoid direct contact with the stimuli during the experiments.

### Experimental Procedures

**Figure 1:**
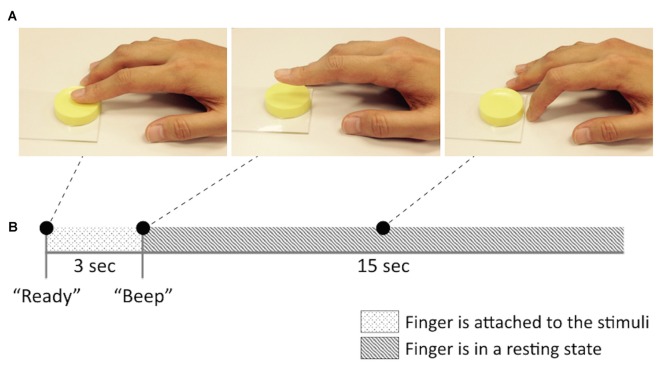
**Brief sketch of the experimental design and the stickiness stimuli. (A)** Finger postures (“Attaching”, “Detaching” and “Resting” from left to right) and **(B)** an example of a single trial in functional magnetic resonance imaging (fMRI) experiment session.

Participants first received a detailed explanation of the experiments. Then, participants conducted a training session (maximum 10 min, 5 min on average) outside the MRI room prior to the behavioral and fMRI experiments. The training session consisted of two tasks. In the first training task, participants were trained to consistently exert pressure on a right index finger while touching the surface of the stimuli. To this end, participants performed several training trials touching the surface of a pressure sensor (A201-100, FlexiForce, MA, USA) with the same level of pressure. The target pressure level was set to 1 N for every participant. The pressure sensor was connected to a microcontroller (ATmega328P on Arduino Uno, Atmel, CA, USA) and the value of the pressure was transferred to the host laptop computer via serial communication. The value of the transmitted pressure recordings was displayed on the laptop monitor as a bar graph. With this visual feedback, participants were able to monitor the amount of the pressure they exerted on the sensor in real time. The second training task was designed to regularize the right index finger motions across participants so as to avoid any unexpected confounding factors from finger-movement differences among the participants. Participants followed the instructions of “Attaching”, “Detaching” and “Resting” finger postures (Figure 1) and practiced a series of these movements until they became familiar with the motions.

Throughout the entire experiment, participants touched all stimuli with their right index finger. Participants conducted two behavioral experiments following the training session. Afterward, participants underwent fMRI experiments. An eye patch was given to participants during both behavioral and fMRI experiments to block visual information.

### Behavioral Experiments

#### Criteria for Selecting Stimuli Sets

Two separate psychophysical experiments were conducted to quantitatively investigate perceptual responses to silicone-based sticky stimuli (Goldstein, 2013). The first experiment employed the method of constant stimuli to measure the absolute threshold of the stimulus prepared for the tactile perception of stickiness. The second experiment employed the magnitude-estimation method to measure the perceived intensity of stickiness sensation. We tried to test all silicone stimuli in each experiment, but it was not feasible considering the total experiment time. Instead, we used a different set of stimuli for each method: 5%, 6%, 7%, 8%, 9%, 10% and 20% stimuli were used for the method of constant stimuli, and 5%, 6%, 7%, 8%, 9%, 10%, 15% and 30% stimuli for the magnitude estimation method. We decided to test silicone stimuli with a catalyst ratio lower than 8% since our pilot study with a separate group of participants (*N* = 6) revealed that an absolute threshold was observed approximately between 7% and 8% ratios. Also, we include 10% stimuli in both tasks, because 10:1 is the standard mixing ratio of the catalyst. We expected to observe a gradual decrease in perceived stickiness as the catalyst ratio increases in the magnitude estimation task. Thus, for stimuli with a catalyst ratio greater than 10%, we included 15% and 30% stimuli for the magnitude estimation test. Meanwhile, since we already expect that the absolute threshold will be defined between 7% and 8% ratio, for the stimuli with a catalyst ratio greater than 10%, we only included the 20% stimulus in the constant stimuli task, which was not tested in the magnitude estimation task.

#### Method of Constant Stimuli

In each trial of the first behavioral experiment, participants touched one of the seven silicone stimuli (5%, 6%, 7%, 8%, 9%, 10% and 20%) with their right index finger and verbally answered “Yes” if the given stimulus felt sticky, or “No” if not. Participants were allowed to touch a given stimulus only once and asked to respond immediately. Each stimulus was presented ten times (70 trials in total) pseudo-randomly.

#### Magnitude Estimation

In the second behavioral experiment, the sham and 7% stimuli were initially presented to participants as references. Previous psychophysical studies have selected a midrange exemplar as a reference for the magnitude estimation test (Eadie and Doyle, 2002; Weismer and Laures, 2002). The result from the pilot experiment (see above) showed that the 7% stimulus was the midrange stimulus among all the silicone stimuli. Participants touched the two references with their right index finger, one at a time starting with the sham stimulus. They were informed that the intensity values of stickiness were 0 and 70 for the sham and 7% stimuli, respectively, where the intensity values were arbitrarily assigned for quantification in our experiment. After this initial calibration, participants performed the trials of magnitude estimation. In each trial, participants first touched the two reference stimuli, followed by experiencing one of the eight stimuli (5%, 6%, 7%, 8%, 9%, 10%, 15% and 30%), and verbally reported the perceived intensity of stickiness of the given stimulus. Participants were instructed to report the estimated intensity value following their own internal scale and informed that there was no maximum limit for the intensity. Each stimulus was presented twice per participant (16 trials in total).

### fMRI Experiments

As this study aimed to find brain regions underlying the tactile perception of stickiness, our investigation focused on the brain responses at the threshold of stickiness perception. Since our pilot study indicated that tactile stickiness was perceived with the stimuli with less than or equal to the catalyst ratio of 7%, we selected the 5% and 6% stimuli, including the 7% stimulus in the test set. Among the stimuli higher than 7%, we chose the 8% and 30% stimuli, which corresponded to the minimum and maximum catalyst ratios, respectively. The 10% stimulus relating to the standard catalyst ratio for PDMS was also added to the test stimulus set. Lastly, the acrylic sham stimulus was utilized for presenting a non-sticky stimulation. To sum up, the 5%, 6%, 7%, 8%, 10% and 30% silicone stimuli as well as the acrylic sham stimulus were used for fMRI experiments to investigate neural responses to the stimuli with different intensities of stickiness.

Participants underwent two scanning sessions and T1 structure images were taken between the sessions. During the functional image acquisition session, participants were comfortably laid in a supine position while holding their right hand down on the MRI bed in a pronation position. They wore a MRI-compatible headphone to listen to the instructions during the experiment. The participants’ heads were fixed to prevent movement artifacts by inserting two foam cushions into the space between the head and the head coil. An event-related paradigm was adopted in our experiment. The procedure for each trial is depicted in Figure 1. The stimulus presentation was carried out manually by an experimenter in the MRI room. Before a stimulus was given, participants were relaxed with the “Resting” finger position. Then, when participants heard the verbal instruction of the “Ready (“Jun-bee” in Korean)”, they attached their right index finger to the given stimulus and maintained the pose for 3 s until they heard a brief beep sound indicating for them to stop. After participants detached their finger from the stimulus at the beep sound, they stayed in the “Resting” posture again for 15 s until the next trial. Each of the 7 stimuli was presented 10 times in a random order, so that a single scanning session consisted of 70 trials. At the beginning of each session, there was a 6-s interval and, thus, each session took approximately 21 min.

### Data Acquisition and Preprocessing

fMRI experiments were performed on a 3T MRI scanner (Magnetom TrioTim, Siemens Medical Systems, Erlangen, Germany) with a standard 12-channel head coil. Functional images were acquired using blood-oxygen-level-dependent (BOLD) sensitive gradient-echo-based echo planar imaging (GE-EPI; TR = 3000 ms, TE = 30 ms, Flip angle = 90°, FOV = 192 mm, Slice thickness = 3 mm, and Voxel size = 2 × 2 × 3 mm^3^) with 47 slices that cover the whole cerebrum. To obtain T1-weighted anatomical images from each participant, a 3D magnetization-prepared gradient-echo (MPRAGE) sequence was used (TR = 1900 ms, TE = 2.48 ms, Flip angle = 9°, FOV = 200 mm, and Voxel size = 0.8 × 0.8 × 1.0 mm^3^). Functional images were preprocessed using SPM8 (Wellcome Department of Imaging Neuroscience, London, UK), which was composed of realignment, slice-timing correction, co-registration, spatial normalization to the Montreal Neurological Institute (MNI) template, and smoothing with a 4-mm full-width-half-maximum (FWHM) isotropic Gaussian kernel.

### Data Analysis

We excluded three participants from the data analysis. While two of them (Subjects 10 and 12) were eliminated because their functional image data was significantly contaminated with noise, another participant (Subject 8) was eliminated due to his abnormal behavioral response which was determined to be an outlier. Specifically, during the magnitude-estimation task, we first transformed all participants’ behavioral responses into *z*-scored values for each stimulus and then set upper/lower fences by adding three folds of the interquartile range (IQR) to the third quartile or by subtracting it from the first quartile. The outlier was defined as the value outside the boundary (Wilcox, 2009). We multiplied the IQR by 3 instead of 1.5 to exclude extreme outliers only (Norris et al., 2014). The behavioral response of one participant was identified as an outlier for the 5% and 7% stimuli. As a result, behavioral and functional data analyses were performed on 9 participants out of 12 in total.

The behavioral data from the method of constant stimuli was analyzed to estimate the absolute threshold of stickiness perception. A psychometric function based on a cumulative Gaussian distribution was fitted to each participant’s behavioral response using the maximum likelihood method. The absolute threshold for each participant was defined as the value at which the stickiness perception could be detected with a 50% chance (Goldstein, 2013).

Analysis of the data from the second behavioral experiment examined differences in the magnitude-estimation responses among stimuli. To this end, we first centralized the magnitude- estimation data of each participant by subtracting the mean value from the original data. Then, the one-way analysis of variance (ANOVA) test followed by the *post hoc t*-test (Tukey-Kramer method) was applied to the mean-corrected data for evaluating a statistical difference between the stimuli.

The functional image analysis was performed using the GLM in SPM8 with a canonical hemodynamic response function and a 128-s high-pass filter to estimate BOLD responses to each stimulus. The moment at which participants detached their finger from the stimuli was set to be an event because the perception of stickiness generally occurs when the skin is stretched by adhesive substances (Yamaoka et al., 2008). We used a different regressor for each stimulus, including the sham stimulus. Since brain regions responsible for processing tactile perception of stickiness were little-known, we employed whole-brain contrasts rather than examining a specific region of interest (ROI). We derived the statistical significance of our study from the second-level analysis, which was implemented by a full factorial design based on a random effect model (Ashby, 2011). Here, the random factor was the subjects and the fixed factor was the tactile stimuli. Significant voxel clusters were identified (*p* < 0.005 (uncorrected) and cluster-extents > 50 voxels) and the coordinates of these clusters were marked according to the MNI space. Using the automated anatomical labeling (AAL) toolbox (Tzourio-Mazoyer et al., 2002), we not only defined the labels of the activated clusters in the SPM, but also subdivided the subcortical regions.

Finally, we performed a correlation analysis between the maximum BOLD signal amplitudes and the intensity of stickiness perception where the perceptual intensity was estimated from the magnitude estimation task performed outside the fMRI scanner. First, we set the activated regions determined by the GLM analysis to be ROIs. Then, we utilized the Marsbar toolbox for estimating absolute maximum BOLD amplitudes of each voxel within a single ROI in response to each stimulus (Brett et al., 2002). Then, the maximum BOLD response of each ROI was obtained by averaging the maximum BOLD amplitudes of all the voxels included in the ROI. A linear regression analysis was used to measure a correlation between the maximum BOLD response and the intensity of stickiness perception such that:

(1) $$y_{i}\;=\;\beta_{1}x_{i}+\varepsilon_{i}$$

where i indicates ith observation, *y*_i_ is the maximum BOLD amplitude, *β*_1_ is a slope parameter, *x*_i_ is a value from the mean-corrected magnitude estimation, and *ε*_i_ is a residual of the model (Motulsky, 2010). In our study, the total number of i was 63, i.e., 9 (the number of subject) × 7 (the number of silicone stimuli in fMRI experiments).

## Results

### Behavioral Responses to Stimuli

#### Method of Constant Stimuli

**Figure 2:**
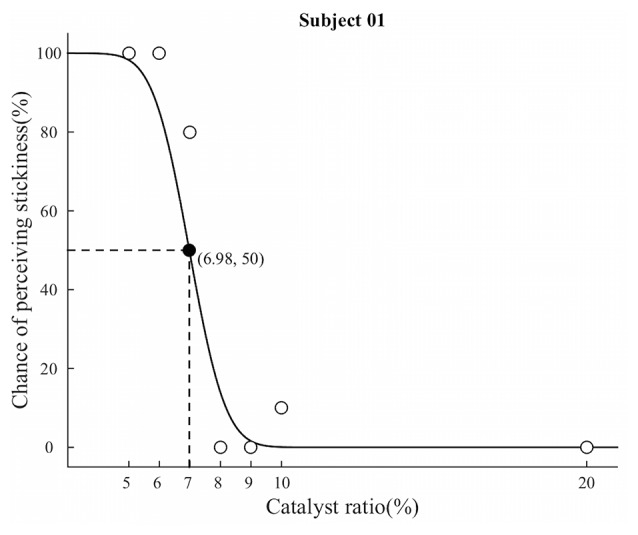
**Results from the method of constant stimuli experiment.** The graph shows a representative result from a participant. White circles indicate the chance of perceiving stickiness of each silicone stimuli, while black circle indicates the absolute threshold determined by the maximum likelihood method. The absolute threshold was determined at between the 7% and 8% ratio stimuli. Based on this observation, we categorized seven different levels of stickiness of stimuli into two distinct groups: “supra-threshold” includes three stimuli with less than or equal to a 7% catalyst ratio; and “Infra-threshold” includes stimuli with greater than an 8% catalyst ratio.

The possibility of perceiving sticky feeling across participants was greater than 0 for all the stimuli (Supplementary Table 1). The behavioral data analysis with the method of constant stimuli revealed the absolute threshold of our silicone-based stimuli for tactile perception of stickiness. The mean absolute threshold across participants was a 7.47% catalyst ratio (SD = 1.31%), and the average standard deviation for cumulative Guassian distribution was 1.03 (SD = 0.42). Figure 2 illustrates a representative psychometric function in a single participant. Participants perceived a sticky feeling almost every time (98.89%) when they touched the stimulus with the 5% catalyst ratio, and the detection rate for stickiness decreased in a nonlinear fashion as the stimulus contained more catalyst.

#### Magnitude Estimation

**Figure 3:**
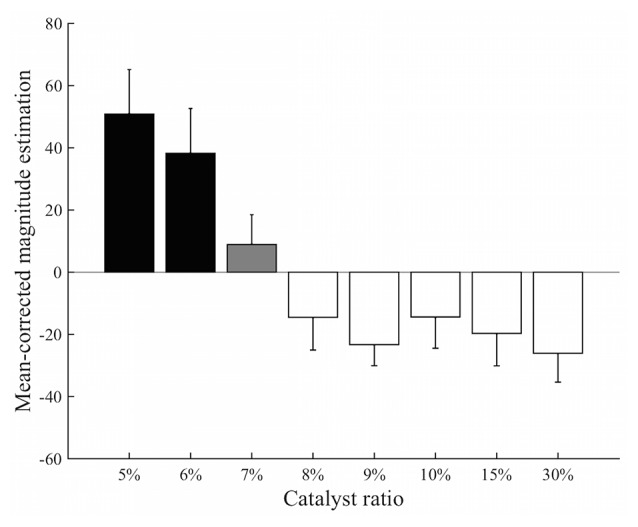
**Results from the magnitude estimation experiment.** The *post hoc* analysis classified eight different levels of stickiness of stimuli into three distinct groups: 5% and 6% stimuli (black); 7% stimulus (gray); and the stimuli containing greater than an 8% catalyst ratio (white). Perceived intensity of the 7% stimulus was significantly different from those of the 5% and 6% stimuli and from those of the 8%–30% stimuli (*p* < 0.0001). There was no significant difference within each group (*p*s > 0.05).

The estimated values of perceived stickiness across participants were all greater than 0 (Supplementary Table 2). The mean-corrected magnitude estimation for different stimuli showed a decrease in the estimated intensity of stickiness as the catalyst ratio increased (Figure 3). The one-way ANOVA test revealed that perceived intensities of stickiness were significantly different across the stimuli (*F*_(7,64)_ = 66.31, *p* < 0.0001). The *post hoc t*-test showed that perceived intensity of the 7% stimulus was less than those with the 5% and 6% stimuli but greater than those with the 8%, 9%, 10%, 15% and 30% stimuli (*p*s < 0.0001 for all). Meanwhile, no significant difference of the perceived intensity of stickiness was found by the *post hoc t*-test between the 5% and 6% stimuli (*p* = 0.24) or between the stimuli with 8% or greater catalyst ratio (*p*s > 0.33 for all).

To confirm that the distinction between the 7% stimulus and the 5% and 6% stimuli was not resulted from the fact that the 7% stimulus was used as a reference, we conducted an additional ANOVA test and a *post hoc* *t*-test on the data of the method of constant stimuli task. The result again showed a similar effect: the chance of perceiving stickiness from the 7% stimulus (Mean = 68.89%, SD = 29.34%) was clearly different from the 5% (Mean = 98.89%, SD = 3.33%) and 6% (Mean = 97.78%, SD = 4.41%) stimuli (ANOVA: *F*_(6,56)_ = 61.08, *p* < 0.0001; *t*-test: *p* = 1.00 (5% vs. 6%), *p* = 0.0062 (5% vs. 7%), and *p* = 0.0095 (6% vs. 7%)).

#### Subdivision of Silicone Stimuli

According to the result of the method of the constant stimuli, the stimuli set were divided into two groups, which were either above or below the mean absolute threshold value (7.47%). The result of the magnitude estimation task also showed that the 7% stimulus was clearly distinguished from the 8%, 9%, 10%, 15% and 30% stimuli. Taken these together, we segmented the silicone stimuli into two groups. The “*Supra-threshold*” group, consisting of the 5%, 6% and 7% stimuli, was likely to evoke the perception of stickiness in participants, whereas the “*Infra-threshold*” group, consisting of the stimuli with 8% or more catalyst ratio, was not likely to induce a sticky sensation. Although the stimuli in the *Infra-threshold* group did not evoke the perception of stickiness very often, most participants felt sticky from them once or more (Supplementary Figure S1, Tables 1, 2). The acrylic sham stimulus was dubbed as “*Sham*” for convenience. This grouping of stimuli was used in the functional data analysis to find the brain regions involved in perceiving stickiness.

### Brain Responses to Stimuli

**Figure 4:**
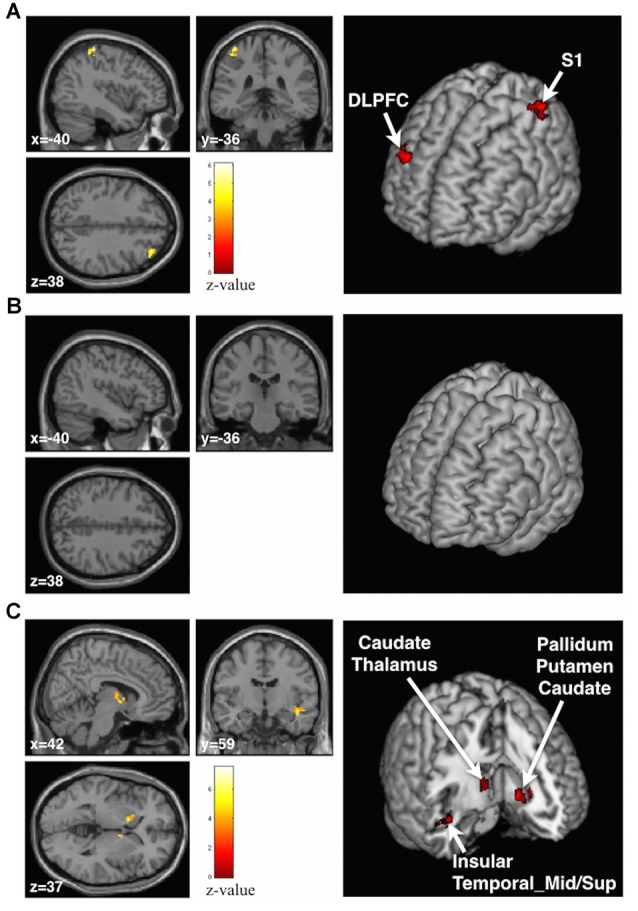
**Anatomical planes (Left) and 3D rendering image (Right) of the brain with significant clusters identified by the group general linear model (GLM) analysis. (A)** At the *Supra-threshold* vs. *Sham* contrast, contralateral postcentral gyrus and ipsilateral dorsal-lateral prefrontal cortex areas were activated. **(B)** No activation was found in the *Infra-threshold* vs. *Sham* contrast. **(C)** At the *Supra-* vs. *Infra-threshold* contrast, the basal ganglia area, insula and middle and superior temporal gyrus areas were activated.

We examined the BOLD effect of the stickiness perception by the *Supra-threshold* vs. *Sham* contrast and found that the contralateral postcentral gyrus (BA 1–3, S1) and ipsilateral middle frontal gyrus (BA 9, dorsolateral prefrontal cortex (DLPFC)) were significantly activated when participants felt stickiness in their index finger (Figure 4A, Table 1). In the Talairach space coordinates, the maximum activation was located at *x* = −42, *y* = −38 and *z* = 64 for S1, and *x* = 34, *y* = 40 and *z* = 36 for DLPFC. On the other hand, no significantly activated brain region was found by the *Infra-threshold* vs. *Sham* contrast (Figure 4B, Table 1). The analysis of the *Supra-* vs. *Infra-threshold* contrast identified three significant clusters (Figure 4C, Table 1). The first cluster was located at the contralateral basal ganglia region, including pallidum, putamen and caudate (Talairach space coordinates of the maximum activation: *x* = −12, *y* = 10 and *z* = −2). The second cluster was placed at the ipsilateral basal ganglia region, including the caudate and thalamus regions (the maximum activation coordinate: *x* = 8, *y* = 0 and *z* = 0). The third cluster was located in the brain regions including the insula as well as the superior and middle temporal cortices (the maximum activation coordinate: *x* = 44, *y* = −10 and *z* = −16).

**Table 1:** **Activated clusters from group general linear model (GLM) analyses**.

Brain regions	Side	Cluster size	MNI coordinate			*T*	*Z*
			*X*	*Y*	*Z*
**Supra-threshold > Sham**
Postcentral	L	55	−42	−38	64	6.13	3.63
			−36	−34	56	3.83	2.81
Frontal_Mid (DLPFC)	R	57	34	40	36	6.07	3.62
			40	34	42	3.81	2.80
**Infra-threshold > Sham**
No activated cluster was found
**Supra- > Infra-threshold**
Pallidum, Putamen, Caudate	L	68	−12	10	−2	7.52	3.98
			−18	16	0	4.24	2.98
Caudate, Thalamus	R	57	8	0	0	6.34	3.69
			8	−4	8	4.52	3.10
Insula, Temporal_Mid, Tempora_Sup	R	50	44	−10	−16	6.25	3.67
			40	−12	−8	5.12	3.32
			54	−12	−14	4.82	3.21

*(p < 0.005 uncorrected, cluster size > 50). Side indicates hemisphere (R = right, L = left), cluster size indicates N voxels, T indicates peak t-values, Z indicates peak z-values. Entries without a brain region label indicate the sub-peak within the cluster named above*.

### Correlations Between the Perceived Intensity of Stickiness and BOLD Responses

**Figure 5:**
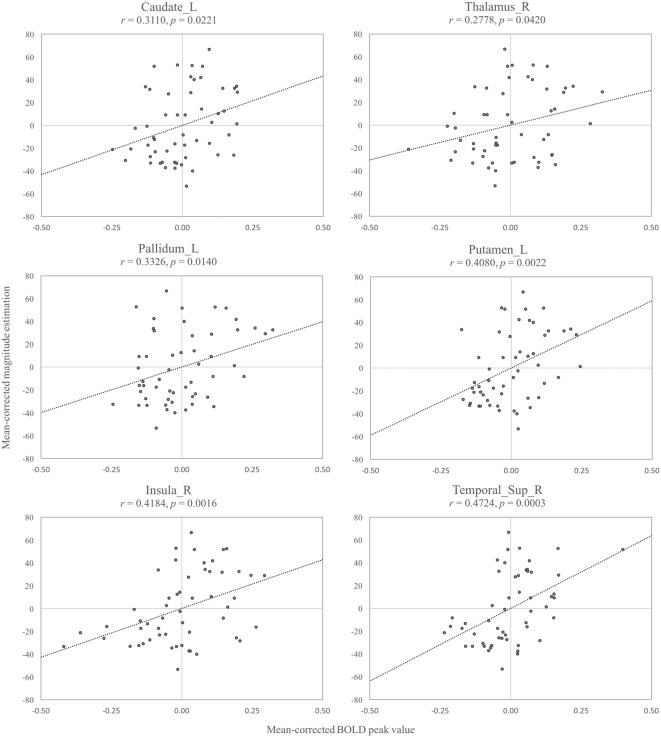
**Among the eight region of interest (ROI) regions that were activated in the *Supra-* vs.* Infra-threshold*, six regions showed significant relationships between the mean-corrected blood-oxygen-level-dependent (BOLD) peak values and the mean-corrected magnitude estimation values (*p*s < 0.05): left caudate (Caudate_L), right thalamus (Thalamus_R), left pallidum (Pallidum_L), left putamen (Putamen_L), right insula (Insula_R), right superior temporal cortex (Temporal_Sup_R)**.

We further investigated how the perceived intensity of stickiness, that was measured through the magnitude estimation task, was related to the activation level in the specific brain regions. We made ROIs by circumscribing the regions that showed a significant result in the *Supra-* vs. *Infra-threshold* contrast. The linear regression analysis between the mean-corrected maximum BOLD and the mean-corrected magnitude estimation showed that, among eight activated areas (pallidum, putamen, contralateral caudate, ipsilateral caudate, thalamus, insula, superior temporal cortex and middle temporal cortex), six areas, all but the ipsilateral caudate (*r* = 0.19, *p* = 0.15) and middle temporal cortex (*r* = 0.10, *p* = 0.48), exhibited significant correlations (*r*s > 0.28, *p*s < 0.05 for all Figure 5). All six brain regions showed a positive relationship between the maximum BOLD response and the perceived intensity of stickiness.

We applied the same correlation analysis for the two brain regions, contralateral S1 and ipsilateral DLPFC, which were activated in the *Supra-threshold vs. Sham* contrast. However, we did not find significant correlations between the BOLD responses of these two areas and the perceived intensity of stickiness (*r*s < 0.06, *p*s > 0.66).

## Discussion

The objective of the present study was to find neural correlates of the tactile perception of stickiness using fMRI. To achieve our goal, we presented participants with silicone-based sticky stimuli to induce tactile feelings of stickiness with different intensities. Behavioral responses from the participants demonstrated that the silicone stimuli could be divided into the *Supra-* and *Infra-threshold* groups based on the absolute threshold of inducing the stickiness perception, that is, the *Supra-threshold* stimulus for clearly evoking perception of stickiness and the *Infra-threshold* stimulus for not. The analysis of the fMRI data revealed that contralateral S1 and ipsilateral DLPFC were significantly activated in the *Supra-threshold* vs. *Sham* contrast, while no significant activation was found in the *Infra-threshold* vs. *Sham* contrast. Moreover, the bilateral basal ganglia, ipsilateral insula cortex, and superior and middle temporal cortex were activated in the *Supra-* vs. *Infra-threshold* contrast. To the best of our knowledge, our study is the first to reveal neural correlates of the perception of tactile stickiness in humans.

### Behavioral Responses in Two Psychophysics Experiments

From the two behavioral experiments, we could observe several key aspects of the perceived stickiness induced by our silicone-based stimuli.

First, the perceptual threshold for stickiness across the participants was determined at a 7.47% catalyst ratio. This result indicates not only that the silicone stimuli could perceptually be divided into two groups, i.e., the *Supra-* and *Infra-threshold* groups, but also shows that our stimulus set could capture the threshold at which participants began to feel sticky.

Second, according to the magnitude estimation, the stimulus with the 7% catalyst ratio had a different intensity of stickiness perception from the 5% and 6% stimuli. One can suspect that using the 7% stimulus as a reference might affect the result. It is possible that the 7% stimulus, which aroused the tactile sensation of stickiness with approximately a half chance, might confuse participants to evaluate the relative intensities of stickiness of other stimuli. However, we intended to have participants estimate the perceived intensity of stickiness of each stimulus compared to the one with no sticky sensation (i.e., Sham) or the one with the midrange stimulus (i.e., the 7% stimulus). The result of the magnitude estimation task also suggests that participants could evaluate the relative stickiness intensities of all presented stimuli without problem. Moreover, the distinction between the 7% stimulus and the 5% and 6% stimuli was also observed in the *post hoc t*-test on the data from the method of constant stimuli. Considering that there was no reference stimulus in the method of constant stimuli task, it is plausible that the 7% stimulus naturally evoked a different perception of stickiness than the 5% and 6% stimuli.

Lastly, the perceived stickiness of the *Infra-threshold* stimuli was not the same as of the *Sham* stimulus that was made of an acrylic material and used to provide the tactile condition of a non-sticky feeling. Although the *Infra-threshold* stimuli failed to generate an apparently sticky feeling, the average behavioral scores for these stimuli in the method of constant stimuli and the magnitude-estimation tests were greater than 0 (Supplementary Tables 1, 2). In the constant-stimuli test, the score of 0 indicates that participants had no feeling of stickiness for all trials, while in the magnitude-estimation test, 0 was equal to the intensity of stickiness of the *Sham* stimulus. Taken together, we can speculate that the *Infra-threshold* stimuli evoked a tactile perception similar to stickiness, but the sensation was too weak to be considered as a sticky feeling.

One drawback of the stimulations employed in the current study is that the physical intensity of stickiness of each silicone stimulus is unknown, so the study was unable to examine the changes in perception of stickiness according to the physical intensity of stickiness. We attempted to measure the physical intensity of stickiness of our silicone stimuli in a follow-up investigation, but no currently available stickiness measurements, such as the peel-strength test, could measure it properly. Hence, it should be noted that the entire analysis in our study was based on the *perceived* intensity of stickiness, not on the *physical* one.

### Brain Responses in the *Supra-Threshold* vs. *Sham* and *Infra-Threshold* vs. *Sham* Contrasts

Contralateral S1 and ipsilateral DLPFC, the two significantly activated regions in the *Supra-threshold* vs. *Sham* contrast, may be involved in the tactile perception of stickiness. Even though both the *Supra-* and *Infra-threshold* stimuli were made of the same silicone substance, only the *Supra-threshold* vs. *Sham* contrast revealed significant activities in the two brain regions. Thus, it is plausible to attribute the activation of contralateral S1 and ipsilateral DLPFC to the perception of stickiness from the stimuli, not to the perceptual differences of the two materials (i.e., the silicone and the acryl).

S1 has been reported to be involved in tactile information processing in a number of fMRI studies (Servos et al., 2001; Pleger et al., 2003, 2006; Hlushchuk and Hari, 2006; Schaefer et al., 2006; Kim et al., 2015). In particular, S1 is well known to participate in the process of discriminative somatosensory perception (Jiang et al., 1997; Schnitzler and Ploner, 2000; Timmermann et al., 2001). As for the relationship between S1 and stickiness sensation, most previous studies documented the role of S1 in the perception of frictional forces. For instance, it was reported that anesthesia of S1 led to failure of frictional sensation evoked by a grip (Brochier et al., 1999). It was also suggested that humans can grip an object because S1 integrates the information from the tactile afferents of discrete frictional senses (Johansson and Cole, 1992). In addition to those previous studies on the involvement of S1 in the perception of friction forces, our study revealed that S1 was also involved in the tactile perception of stickiness in humans, which has hitherto been unexplored.

The activation in DLPFC has been implicated in many different roles in cognitive processing (Ridderinkhof et al., 2004; Rubia and Smith, 2004; Pleger et al., 2006; Uddin, 2014). Among various interpretations, DLPFC, with the connection to the parietal cortex, was known to process higher-order somatosensory information (Wood and Grafman, 2003). Furthermore, Navratilova and Porreca (2014) attributed DLPFC activity to the reward mechanism by a relief from an aversive state. Collectively, the previous studies imply that the perception of stickiness evokes a complex feeling, rather than simple tactile sensation. With a high probability, the sticky feeling can arouse a negative emotion to people. Thus, it is plausible that the perception of stickiness can induce emotions such as a relief from aversive states, which might be reflected in the activation of DLPFC in our study.

### Brain Responses in the *Supra-* vs. *Infra-Threshold* Contrast

By contrasting brain responses to the *Supra-* vs. *Infra-threshold* stimuli, we investigated brain regions involved in the perception of different intensities of stickiness. Since all of the stimuli were made of the same silicone material in which consistent perception of stickiness relied only on the catalyst ratio, it can be assumed that the *Supra-* vs. *Infra-threshold* contrast points to the brain regions involved in perceiving different intensities of stickiness. These brain regions broadly included two areas: (1) subcortical areas; and (2) insula to temporal cortex.

It is noteworthy that the activated regions were distributed extensively in subcortical areas (i.e., basal ganglia and thalamus). Of the regions, the activation in basal ganglia and thalamus may reflect the function of the basal ganglia–thalamocortical loop. Traditionally, the motor control aspects of this loop have been of primary interest (Alexander and Crutcher, 1990; Middleton and Strick, 2000), and the role of the loop in processing somatosensory information has been mainly attributed to proprioception (Kaji, 2001). Recent studies, however, have also revealed that the basal ganglia–thalamocortical loop is involved in tactile discrimination (Peller et al., 2006), along the pathway extended from the thalamus to the somatosensory cortex (Vázquez et al., 2013). In this respect, we conjecture that the activation in the basal ganglia and thalamus regions in the *Supra-* vs. *Infra-threshold* contrast may be related to the discrimination of different intensities of stickiness.

Our conjecture is also supported by McHaffie et al. (2005) who argued that the basal ganglia–thalamocortical loop contributes to solving the “selection problem”. Particularly, if a given sensation leads to a consequence of two incompatible systems (e.g., “approach” and “avoid”), the basal ganglia–thalamocortical loop prioritizes information flows that simultaneously enter, and relays it to an appropriate motor output. In this context, tactile information delivered by the silicone stimuli could form an incompatible sensation, which is either above or below the perceptual threshold of stickiness, and the basal ganglia–thalamocortical loop might encode such incompatible sensations differently for effective access of shared sensorimotor resources. Moreover, as the basal ganglia–thalamocortical loop interacts with cortical regions (McHaffie et al., 2005), the judgment for the sensory information at the basal ganglia–thalamocortical loop might be coupled with the activation in cortical levels, as we observed in the *Supra-threshold* vs. *Sham* contrast. Hence, one putative explanation of the activation in the basal ganglia and thalamus may be that the perception of stickiness from the silicone stimuli requires judgment for the sticky sensation in the basal ganglia-thalmocortical loop, and such judgment leads to responses in the cortical region. Yet, this suggestion needs further justification.

The *Supra-* vs. *Infra-threshold* contrast showed an activated cluster spanning from the insula to the temporal cortex as well. Several neuroimaging studies revealed activations in these regions in response to tactile stimulation. While some of them reported the results in the case of presenting the tactile and visual stimuli simultaneously (Banati et al., 2000; Saito et al., 2003; Cardini et al., 2011), Lundblad et al. (2011) observed activations in these regions when subjects performed a tactile discrimination task. In line with those previous reports, our results suggest that the superior and middle temporal cortices as well as insula can be related to the tactile perception of sticky stimuli, presumably for distinguishing delicate differences of the perceived intensity of stickiness.

### Correlation Between Perceived Intensity of Stickiness and BOLD Responses

The result of the *Supra-* vs. *Infra-threshold* contrast indicated that fine perceptual distinction of stickiness might be attributed to the subcortical and cortical areas including the basal ganglia, thalamus, insula and temporal cortices. Hence, we examined a correlation between the estimated intensity of stickiness and the maximum BOLD response in each ROI of these areas. Except for the ipsilateral caudate and middle temporal cortex, all six ROIs showed a positive relationship between the behavioral response and BOLD signal changes, implying that the perception of stronger stickiness accompanies higher BOLD activation in these brain regions.

To confirm whether these correlations are specific to the subcortical areas, we additionally applied the same analysis to the two activated regions in the *Supra-threshold* vs. *Sham* contrast: the contralateral S1 and the ipsilateral DLPFC. The analysis showed no significant correlation between the activation in the two cortical regions and the behavioral responses, thereby supporting that the activation of the subcortical areas may reflect the perception of different intensities of stickiness.

### Limitations and Future Work

The present study has some limitations. With regards to the experiment, we did not record the behavioral responses from participants about the perceived intensity of stickiness during the fMRI scanning. We made our participants focus more on stimuli and minimize extra movements during the response in order to avoid imaging artifacts. However, although we were able to find the relationship between the perceived intensity of stickiness, which was acquired outside the scanner, and the neural responses, which were acquired inside the scanner, it still remains unclear whether participants felt exactly the same tactile perception from the stimuli during the fMRI experiment. Moreover, we trained our participants in a sitting posture and were not able to measure the finger force during scanning where participants lied inside the scanner. This might make one doubt whether the participants performed finger movements well in the fMRI experiment as they had been trained in a different posture. However, the movement was confined to the right index finger, which would be minimally affected by the body posture, and we confirmed that participants fully acquainted with the pressure. Thus, we conjecture that our participants performed well in the experiment as they were trained. The small number of subjects could also have hindered making our findings firm enough. We were not able to recruit more participants in our experiment due to the limitation in silicone-based sticky stimuli. An additional experiment was not available since it was impossible to produce exactly same silicone stimuli set and the silicone-based sticky stimuli naturally degraded over time. This limitation poses a challenge in research of tactile perception with sticky stimuli and may underline the necessity of developing a more sustainable stickiness stimulation system such as the one using air dynamics (Yamaoka et al., 2008). Lastly, there is incongruity in the interpretation of BOLD activation in the subcortical areas. For example, if the judgment for the intensity of the sticky sensation activates the basal ganglia and the thalamus, similar effects should be observed in the *Supra-threshold* vs. *Sham* and the *Infra-threshold* vs. *Sham* contrasts. However, this was not observed in our current study, leaving the exact role of the subcortical areas in stickiness perception still subject to further investigation. Though this study found several brain regions that are putatively responsible for processing the tactile sensation of stickiness, it is necessary to clarify and confirm the result with more sophisticate methods in follow-up studies.

The present study only reveals the direct brain responses when people perceive a tactile feeling of stickiness. This is the first attempt to measure brain activity related to the perception of stickiness, but the topic should be more extensively investigated to extend our knowledge on human tactile perception. Particularly, for the comprehensive understanding of the four tactile perception dimensions and their neural mechanisms, it is necessary to make an endeavor to explore all these tactile dimensions together and to investigate associated neural systems thoroughly. We envision that future work will uncover the detailed neural mechanisms underlying human tactile perception.

## Author Contributions

All co-authors contributed to the article as follows: JY participated in all aspect of the work, analyzed the data and wrote the manuscript. JK collected and analyzed the data and edited the manuscript. JR took part in the experiment as well as in the fMRI data acquisition. J-YP directed literature review and suggested a perspective on this study. S-CC provided theoretical bases. S-PK oversaw the study and managed every part of research.

## Conflict of Interest Statement

The authors declare that the research was conducted in the absence of any commercial or financial relationships that could be construed as a potential conflict of interest.
