# Supplementary material for: Human Brain Activity Related to the Tactile Perception of Stickiness
Source: Front Hum Neurosci. 2017 Jan 20;11:8. doi: 10.3389/fnhum.2017.00008 (PMC5247468; doi:10.3389/fnhum.2017.00008)
Supplement: Supplementary file 1 [file SupplementaryMaterials.docx]

Supplementary Material

Human brain responses correlated with the tactile perception of stickiness

Jiwon Yeon, Junsuk Kim, Jaekyun Ryu, Jang-Yeon Park, Soon-Cheol Chung, and Sung-Phil Kim^*^

*** Correspondence:** Sung-Phil, Kim: spkim@unist.ac.kr

# Supplementary Figure

#
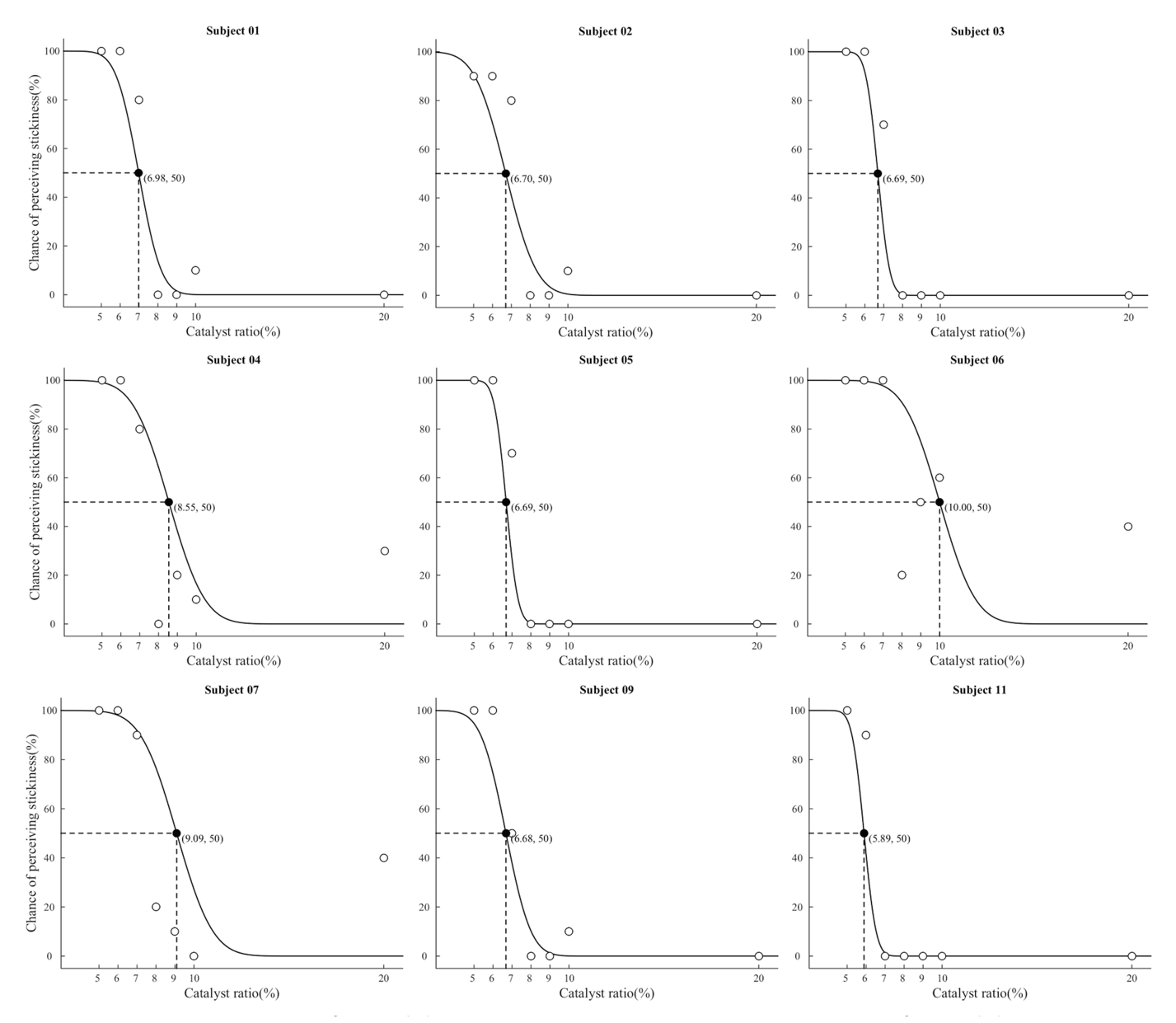


**Supplementary Figure S1.** Psychophysics fitting results of each participant’s responses for the method of constant stimuli task. Subject 8, 10, and 12 were excluded since they were revealed as outliers in the magnitude estimation test. The y-axis indicates the possibility of perceiving stickiness obtained from the participants responds, and the x-axis shows the catalyst ratio of the silicone stimuli. In each graph, white circles display the participants’ response of perceiving stickiness for each silicone stimuli. We can fit psychometric functions to each participants’ response based on the cumulative Gaussian distribution using the maximum likelihood method. The black circles indicate the fitted absolute threshold of perceiving stickiness for the participants, and the numbers are the coordinates for the black circles.

# Supplementary Tables


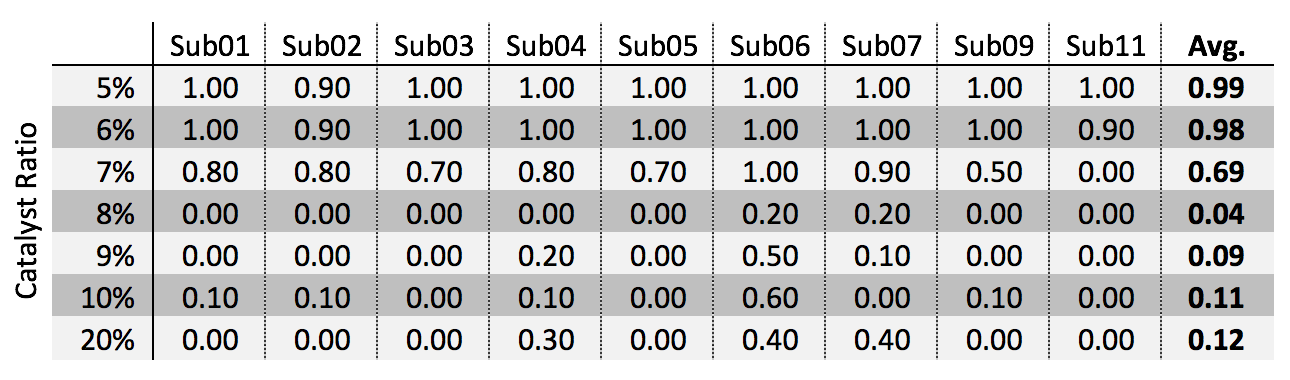


**Supplementary Table 1.** The probability of perceiving stickiness from each silicone stimulus in individual participants in the method of constant stimuli test.


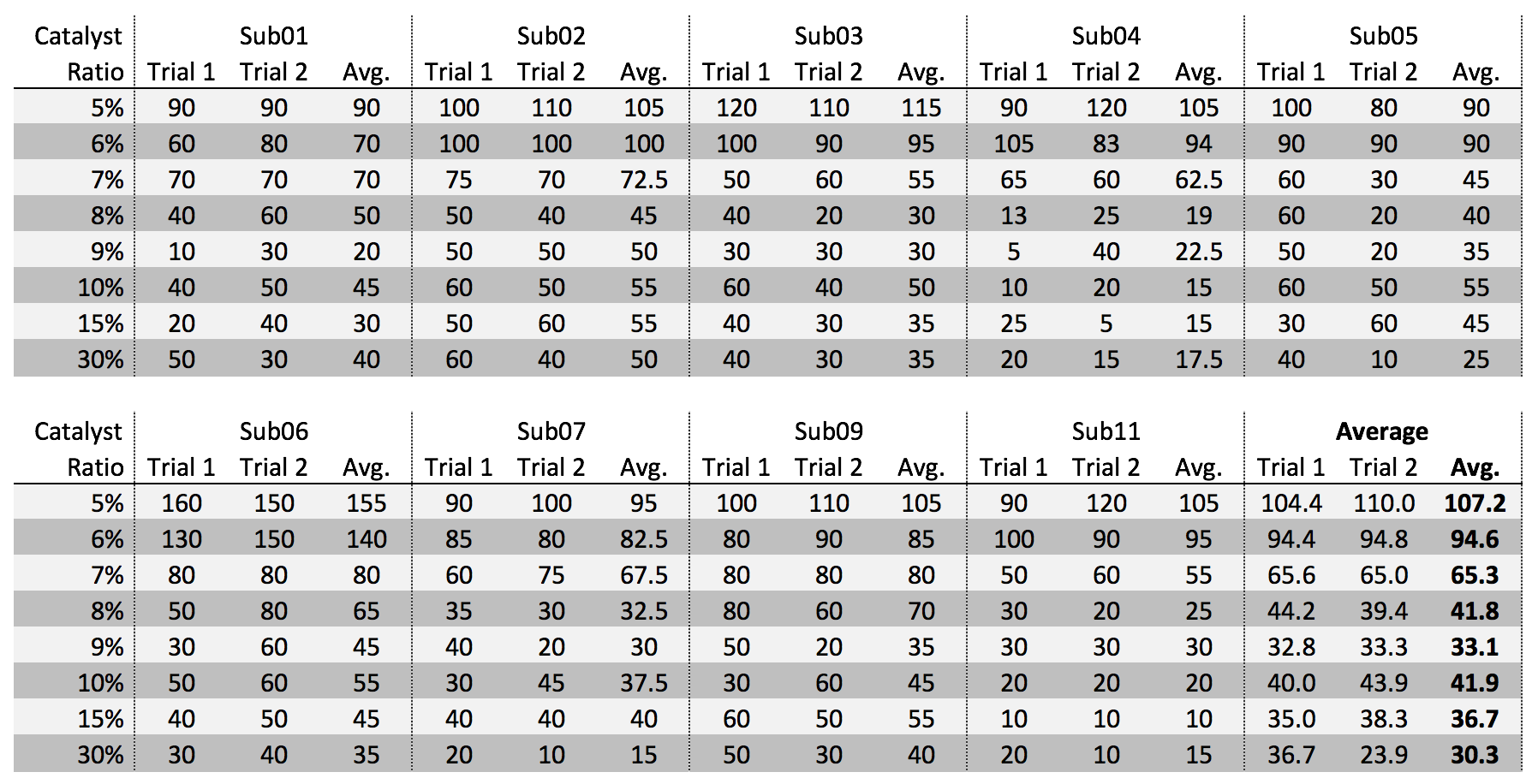


**Supplementary Table 2.** Numerical scores for the stickiness of each silicone stimulus estimated by individual participants in the magnitude estimation test. Note that the stimulus with the 7% catalyst ratio and the acrylic sham stimulus were dictated to participants as references with scores of 70 and 0, respectively.
